# Developing an Intervention for Safe Hospital Insulin Use for Older Adults With Diabetes Undergoing Surgical Admission (SHINE Study): A Co‐Design Study

**DOI:** 10.1111/hex.70622

**Published:** 2026-03-04

**Authors:** Christina Lange Ferreira, Hellena Habte‐Asres, Jyothish Govindan, Dionne Mytton, Angus Forbes, Kirsty Winkley

**Affiliations:** ^1^ Care in Long Term Conditions, Florence Nightingale Faculty of Nursing, Midwifery & Palliative Care King's College London London UK; ^2^ Diabetes & Endocrinology Wye Valley NHS Trust Hereford Herefordshire UK

**Keywords:** co‐design, diabetes mellitus, inpatient safety, insulin, older adult

## Abstract

**Background:**

Insulin errors in inpatients with diabetes occur frequently during surgical admissions. Older adults have higher risks. There is a need for service user involvement in developing complex interventions to improve insulin safety in hospitals.

**Objective:**

To develop a logic model for a system‐based safety intervention to support safer insulin use for older adults with diabetes undergoing surgical hospital admission.

**Methods:**

A co‐design approach combining systems thinking and design thinking methods was employed. Purposive sampling was employed. Older adults with diabetes and multiprofessional staff working across perioperative care were involved as co‐designers and decision makers throughout the iterative process of intervention development. An initial exploratory phase included semi‐structured interviews with service users (*n* = 10), hospital staff (*n* = 23) and non‐participatory observations (*n* = 3) which informed subsequent collaboration. Initial findings validated in separate service user and staff workshops and engagement activities. The resulting data were presented at joint stakeholder workshops (*n* = 3) to confirm the final intervention components needed to develop the model. In total, *n* = 10 older adults and *n* = 11 healthcare professionals working across the perioperative pathway participated in the co‐design process between October and December 2024.

**Results:**

Co‐designers were engaged and collaborative. Multiple interacting components at patient, staff and context level identified. Through an iterative process, a co‐designed logic model known as the SHINE (safe‐hospital‐insulin‐use) wheel was constructed, addressing two prioritised areas: *transitions of care* and *right insulin, time and way*. The model also identifies eight actions and twelve separate outputs as components. Solution‐related themes centred around: *it is all so connected; right insulin, right time, right way; safer transitions of care; empowerment; organisation and provision of care; developing and supporting the workforce*. Two prototypes of tools for patient and staff education to support empowerment and increase patient preparedness for hospital admission were developed.

**Conclusion:**

Multiple interacting components influence hospital insulin safety. System‐based, non‐linear safety approaches are required. This co‐design study identified priorities of older adults with diabetes and healthcare professionals to be addressed in system‐wide insulin safety interventions for surgical admissions. Tools developed provide tangible outputs for application in clinical practice.

**Patient or Public Contribution:**

People with diabetes with recent lived experience of surgical hospital admission were integrally involved as co‐designers and decision makers in this study. They contributed towards interpretation and analysis of findings through discussion, feedback and validation in workshops and through means accessible to them. They were decision makers in the identification of priorities for intervention development and in identifying and validating the content of the toolkit developed.

**Abbreviations**
HCP: Healthcare Professional
MRC: Medical Research Council
NHS: National Health Service
RESILIENT Framework: Interacting components in insulin use in hospital Framework
SHINE: Safe Hospital Insulin Use

## 1 Background

Insulin treatment is a common treatment in people with type 2 diabetes and all those with type 1 diabetes. Insulin regimens are heterogeneous in the types of insulin and modes of action (short, intermediate and long acting), insulin timings, complexity and associated technologies (delivery devices and glucose monitoring). Insulin treatments are individualised and tailored to patient need; this lack of standardisation means that managing insulin in hospital is challenging, further compounded by the metabolic effects of illness or surgery. Insulin is considered a time‐critical, high‐risk medication due to its narrow therapeutic window and risk of severe harm in the case of errors [1].

There are significant challenges to managing the multiple components associated with insulin therapy in hospital [2, 3, 4, 5, 6]. The perioperative context is particularly complex, as it requires several transitions of care and input from multiple specialties and clinicians [7, 8]. Insulin adjustments are required in preparation for surgery, and stress‐related hyperglycaemia occurs frequently and is associated with surgical procedures and hospitalisation [8]. Optimal glycaemic control is important to reduce the risk of complications, delayed healing and length of hospital stay [8, 9, 10]. Insulin errors can lead to emergencies such as diabetic ketoacidosis and hyperosmolar hyperglycaemic state, and severe hypoglycaemia [7]. Less acute complications include sub‐optimal glucose variability which can have clinical significance, impact negatively on patient experience and can be anxiety‐provoking for people with diabetes [4, 7, 11].

Several areas of increased risk for insulin error in the perioperative setting have been identified in national reports and in the literature: diabetes and insulin treatment not identified/communicated at initial referral from primary care and at transitions of care [12, 13, 14]; failure to monitor glucose adequately at all stages along the perioperative journey [13, 14, 15], incorrect use of intravenous insulin infusions [13, 14], lack of clinical continuity from the different specialties involved across the perioperative pathway [13], staff knowledge and confidence gaps [12, 13], lack of proactive treatment review in response to out of target glucose levels [6] not prioritising people with diabetes on operating lists and carbohydrate intake issues exacerbated by fasting required for diagnostic or surgical procedures [13]; incomplete or incorrect information about required treatment adjustments pre‐surgery provided to patients pre‐operatively [12].

The majority of hospitalised patients are aged > 65 years [16] with a high proportion of those patients having co‐morbid diabetes [16, 17, 18]. The older hospitalised population is heterogeneous and there are many factors associated with older age that increase their risk of insulin errors. These may include frailty, polypharmacy, and a range of communication, cognitive, physical or nutritional deficits, alongside an impaired ability to self‐manage their insulin [19, 20] However, most studies which have looked at insulin safety in hospital have not focused on the particular needs of this population [5, 6, 11, 12] and thus there is currently limited research and a knowledge gap on the specific needs of hospitalised older adults requiring insulin.

A recent scoping review highlighted the limited involvement of people with diabetes in the development of hospital insulin safety interventions [21] A systematic review of interventions to improve insulin prescribing in hospital also demonstrated a lack of theory‐driven interventions, and low service user involvement [1]. Many safety interventions have focused on specific parts of the insulin process, such as prescribing, administration, self‐administration, IV insulin management and documentation [5, 22, 23, 24]. Others have focused on specific professional groups [25, 26] or on a certain care episode, for instance, ward care or discharge [5, 27].

Given the interconnected nature of managing insulin across hospital services [3] and the safety risks associated with transitions of care [28], more consideration should be given to insulin use across the whole perioperative patient journey of older adults with diabetes in hospital to identify components for intervention development. This requires system‐based approaches to insulin safety. Given the knowledge gap in understanding experiences of older adults with diabetes in surgical inpatient contexts, involving them as co‐designers ensures the intervention development is grounded in lived experience.

## 2 Methods

### 2.1 Design

The findings reported in this paper are from a study to develop an intervention for safer hospital insulin use for older adults living with diabetes, with or without frailty, undergoing a surgical hospital admission (SHINE Study) [29]. Guided by the Medical Research Council/National Institute for Healthcare and Research Framework for developing and evaluating complex interventions [30], the reported study uses an integrated approach of co‐design, based on the design thinking model, which uses a human‐centred approach to understand experiences and build empathy [31, 32, 33, 34] with a system thinking lens, which acknowledges the complexity and interacting components implicated in hospital insulin use [21, 35, 36]. Design Thinking involves three phases: inspiration‐ understanding experiences and challenges and defining the problem; ideation‐ finding solutions and developing prototypes; and implementation, testing and piloting the intervention [31, 37]. We have previously reported the findings of the inspiration phase [38, 39]. This paper reports on the findings from the second phase (ideation), which built from inspiration phase and involved ideating and prototyping solutions to improve insulin safety in hospital for older adults. Reporting adheres to the Guidance for reporting intervention development studies in health research (GUIDED) [40], as signposted in the Medical Research Council Framework for development and evaluation of complex interventions [30].

### 2.2 Study Setting and Participants

#### 2.2.1 People With Diabetes

Participants were recruited from a single National Health Service (NHS) trust in a rural county which serves patients in England and Wales. Older adults with type 1 or type 2 diabetes who had been admitted for, general or orthopaedic surgery, either electively or as an emergency, were eligible for inclusion. Further inclusion criteria were: being able to give informed consent; aged ≥ 65 years; treated with insulin prior to and during hospital admission; having had a history of admission for surgery requiring as a minimum an overnight stay, within 9 months of recruitment (to allow recovery time and to enhance accurate recall of their experiences); and being a fluent English speaker.

#### 2.2.2 Staff

Multiprofessional staff working across the perioperative pathway were eligible for inclusion provided they had worked for a minimum of 3 months at the research site, to ensure they had familiarity with local challenges, processes and systems. Further details regarding hospital site characteristics can be found in Supporting Information (Appendix 1).

### 2.3 Sampling

Purposive sampling was used to target older adults with different types of diabetes, insulin regimens, and levels of frailty, with experience of emergency or elective surgery. Healthcare staff were also sampled to ensure representation of different professional groups and levels of experience, working in different parts of the perioperative care pathway. This approach allowed for inclusion of diverse experiences and perspectives which is key in intervention development [30]. We aimed to include a minimum of six to eight older adults with diabetes and six to eight healthcare professionals in our co‐design ‘ideation' phase of the study, which would be sufficient to achieve the main aims of the co‐design process [41, 42]. The study was advertised through health‐specific social media, research site newsletters and posters displayed in clinical areas; participants were also recruited directly via a retrospective review of admissions by the diabetes team at the site [29]. Eligible participants were screened in person or remotely by the lead researcher (CLF), provided with an information sheet and given time to consider participation and ask questions. Written informed consent was obtained prior to participation. A one‐off £15 voucher was provided to all patients and to staff who were participating in the research outside their usual working hours.

### 2.4 Ethical Approval

National Health Service (NHS) Health Research Authority ethical approval from East Midlands‐Derby Research Ethics Committee (24/EM/0022) and research site approval was gained prior to study commencement.

### 2.5 Data Collection

The study's ideation phase was organised into two parts (A and B). In part A, parallel feedback activities were held with each participant group (staff and patients). In part B, integrated (joint) co‐design workshops were held where staff and patients attended together and contributed jointly.

**Figure 1:**
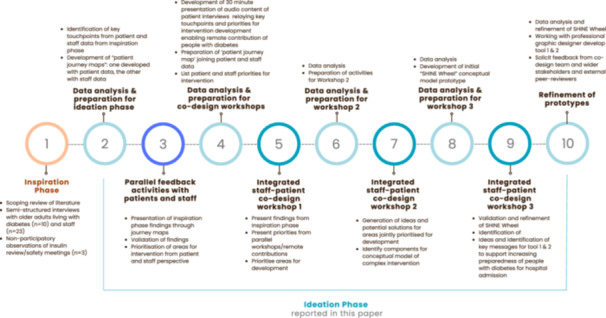
Visual representation of SHINE study interconnected activities.

Figure 1 shows a visual representation of the interconnected sequence of data collection and analysis points during the SHINE Study. All workshops were facilitated by CLF in venues selected for participant convenience. The lead researcher (CLF) was supported by a nursing associate (DM) from the diabetes specialist team at the research site. Workshops were held between October and December 2024. CLF is a female researcher trained in qualitative methods with a clinical background in diabetes nursing.

Table 1 summarises the study data types, collection points, and activities which occurred during the course of the study. Collected data included audio‐recordings of the workshops which were transcribed, sticky notes and electronic whiteboards with participants comments and evaluation forms.

**Table 1:** Summary of SHINE study data types and collection points.

**Data collection point**	**Objective**	**Participants**	**Activities**	**Data types**
**Ideation phase Substage A: Parallel feedback activities with patients and staff (reported in this paper)**				
Workshop for HCP (*n* = 7) Duration of workshop: 2 h	Presenting and validating findings of inspiration phase amongst staff Identification and prioritisation of areas amenable to intervention development	Multiprofessional staff working in hospital/trust	Presentation of patient journey map using illustrations and ‘touchpoints' obtained following analysis of staff interviews (previous study) (Appendix 2) Presentation of quotes to facilitate discussion (from previous study) Dot voting activity for priority ranking (selection of top 6 priorities)	Transcript of audio‐recorded group discussions Sticky notes with comments Patient journey map notes Whiteboard sheet notes with ideas, priority ranking and notes Field notes Evaluation forms
Workshop for people with diabetes (*n* = 1)* Duration of workshop: 1 h	Presenting and validating findings of inspiration phase amongst people with diabetes Identification and prioritisation of areas amenable to intervention development	Older adults with diabetes treated with insulin with recent lived experience of surgical admission at study site	Presentation of patient journey map using illustrations and ‘touchpoints' obtained following analysis of patient interviews (previous study) Presentation of quotes to facilitate discussion (from previous study) Dot voting activity for priority ranking *2 participants cancelled in the 24 h prior to event due to health reasons, opting for remote contribution instead of re‐scheduling F2F event. The single attendee still completed all the workshop activities	Patient journey map notes Transcript of audio‐recorded discussion between patient, CLF and nursing associate supporting event. Sticky notes with comments Whiteboard sheet notes with ideas, priority ranking and notes Field notes
People with diabetes remote contribution (*n* = 9)	Presenting and validating findings of inspiration phase amongst people with diabetes Individual identification and prioritisation of areas amenable to intervention development	Older adults with diabetes treated with insulin with recent lived experience of surgical admission at study site	Postal pack sent to participants which contained instructions and an A3 printout of the ‘patient journey map' (Appendix 3) with a pre‐stamped return envelope. Participants could add comments and were asked to highlight their top 6 priorities for areas amenable to intervention development	Patient journey map notes completed remotely and returned to CLF
**Ideation Phase Substage B integrated staff‐patient co‐design workshops (reported in this paper)**				
Integrated staff‐patient co‐design workshop 1 Presential attendance (*n* = 7) 2 PWD & 5 HCP Duration 2.5 h Remote contribution of people with diabetes through audio content (n = 10)* *The 2 PWD who attended F2F also released their audio contribution from interview	Present findings from inspiration phase Present priorities from parallel activities (ideation phase substage A) Jointly prioritise areas for development	Multiprofessional staff working along the perioperative pathway Older adults with diabetes with recent lived experience could attend face‐to‐face or contribute remotely by releasing audio content from their interviews, highlighting their experiences and priorities for intervention development (having checked portions of interview transcripts to be edited and released and completed prior journey map activity)	30‐min audio presentation of portions from patient interviews to relay “key touchpoints from patient interviews and ensure their voice represented Presentation of patient journey map with challenges as presented by patients and staff Presentation of quotes to facilitate discussion (from previous study) Discussion and voting of joint priorities to take forward to development in workshop 2	Patient journey map notes Transcript of audio‐recorded group discussion Sticky notes with comments and preliminary ideas Whiteboard sheet notes with ideas, comments and priority ranking Field notes Evaluation forms
Integrated staff‐patient co‐design workshop 2 Presential attendance (n = 8) 2 PWD 6 HCP Duration of workshop 2.5 h	Generate ideas and potential solutions for the initial 6 areas jointly prioritised for intervention development Iteratively build conceptual model of components for complex intervention	Multiprofessional staff working along the perioperative pathway Older adults with diabetes with recent lived experience of surgical admission at study site	2 smaller co‐design groups (each with 1PWD) Each group worked on 3 jointly prioritised areas for intervention development (workshop 1) Design thinking tools (Critical items diagram and 2×2 matrix) used to support divergent‐convergent process of idea and solution generation and identification of components for conceptual model Critical items diagram to structure the findings from the early phases and prepare for ideating and experimenting 2×2 Matrix: To categorise and prioritise ideas or identify strategic opportunities and patterns. Ideas that had been identified at previous SHINE study activities were also pre‐printed on laminate cards to ensure wider key player ideas and perspectives included. Example of completed 2×2 matrix in Appendix 4 Supporting Information. Patient and staff journey map printouts and RESILIENT Framework (scoping review findings) were accessible for participants to consult during events Wider facilitated group discussion: each smaller co‐design group presented their perspectives, challenges and solutions and the group identified possible components for conceptual model of intervention	Patient journey map notes Transcript of audio‐recorded group discussion Sticky notes with comments and preliminary ideas Whiteboard sheet with 2×2 matrix notes with ideas, comments and priority ranking Field notes Evaluation forms
Integrated staff‐patient co‐design workshop 3 Presential attendance (*n* = 6) 1 PWD 5 HCP 1 PWD remote contribution to content for tool 1Duration of workshop 2.5 h	Iteratively refine prototype of conceptual model of components for complex intervention. Design of prototypes: Identify content for tools 1 & 2 to support patient empowerment and preparedness for hospital admission	Multiprofessional staff working along the perioperative pathway Older adults with diabetes with recent lived experience of surgical admission at study site	Presentation and discussion of SHINE Wheel conceptual model prototype developed visually by CLF, HH‐A, AF and KW, resulting from all the previous work in SHINE Study. Activities in 2 smaller co‐design groups refining the SHINE Wheel conceptual prototype. The PWD spent time with both groups: Group 1 worked on identification of inputs (resource required) and assumptions (underlying beliefs or conditions); Group 2 worked on identification of outputs & outcomes and ways of monitoring & evaluation for the SHINE wheel conceptual model prototype Group discussion to agree components to include in refined prototypes: SHINE Wheel & tools 1 & 2	Refined SHINE Wheel conceptual model prototype Whiteboard sheet with ideas, comments for specific components/outputs including ideas, comments and content for tool 1 & 2 to be developed Transcript of audio‐recorded group discussion Field notes & Evaluation forms
Feedback	Refinement of prototypes	Study participants	Email, telephone calls, or meetings: feedback to specific questions regarding prototype content, design, relevance, usability	Infographic prototypes (SHINE Wheel, Tool 1 & 2)

#### 2.5.1 Parallel Feedback Activities

Prior to these workshops, interview transcripts from the qualitative interviews (inspiration phase) had been read by CLF and the research team (HH‐A, AF and KW) to identify “touchpoints.” The touchpoints express the significant points that occurred in relation to their insulin experiences during the care journey [42]. These were then re‐phrased as “data driven active sentences” (e.g. *“Not being able to access my insulin”*) and organised into two separate patient journey maps (one developed with staff data and the other with patient data originating from the interviews held in the inspiration phase) (see Supporting Information, Appendices 2 and 3). These maps represented key experiences and impacts and related these to the care context (interactions between patients and healthcare professionals and the care system).

Presentation of the maps to the workshop participants led to further discussions of the touchpoints, allowed for further comments to be added, enabled the validation of findings and provided a list of priorities to improve safety. After several older adults had expressed problems with face‐to‐face workshop attendance, all patient participants were provided with the option to review the journey maps remotely, validate findings, identify interventions and give feedback by post. Post was the participants' preferred method and helped avoid digital exclusion.

#### 2.5.2 Integrated Staff‐Patient Co‐Design Workshops

Three integrated staff‐patient co‐design workshops were then held, each building iteratively upon ideas obtained from earlier workshops.

#### 2.5.3 Integrated Workshop 1

The aim of this first workshop was to present the priorities of each participant group to facilitate discussions, leading to establishing joint/shared priorities for intervention development. A 30‐min audio presentation was presented to stimulate ideation, the presentation used audio extracts of the 10 interviews conducted in the inspiration phase. The presentation followed the patient and used captions to identify the touchpoint themes as the audio played. The presentation was used in the first joint co‐design workshop, alongside the two journey maps which had been presented at the parallel workshops. Participants not attending in person were able to contribute their experiences and priorities for intervention development remotely by post or email.

#### 2.5.4 Integrated Workshops 2 & 3

These workshops encouraged the formation of smaller groups to generate further ideas and solutions, and review and refine prototypes. These small groups then shared their ideas and views with all workshop participants to agree what ideas should be taken forward.

The field notes, transcripts and materials created in each workshop were analysed by the lead researcher (CLF) post‐workshop and then communicated to participants at subsequent workshops to inform focus and promote intervention development.

Any final prototypes and tools which had been developed during the workshops were then circulated to participants via email, face‐to‐face or through phone discussion to verify, review and refine (Table 2).

**Table 2:** Characteristics of SHINE study participants.

People with diabetes participants								
**Study identifier**	**Sex**	**Age (Years)**	**Ethnicity**	**Type of diabetes**	**Duration of diabetes**	**Type of insulin regímen and level of self‐managing prior to admission**	**Type of admission and length of stay in hospital**	**SHINE study activities**
1	Female	77	White	Type 1 diabetes	23 years	Self‐managing multiple daily injections with basal and bolus analogue insulins	Emergency	I, RC, F
2	Male	73	White	Type 1 diabetes	56 years	Self‐managing multiple daily injections with basal and bolus analogue insulins	Elective	I, RC
3	Male	68	White	Type 1 diabetes	40 years	Self‐managing multiple daily injections with basal and bolus analogue insulins	Elective	I, RC
4	Female	72	White	Type 1 diabetes	61 years	Self‐managing multiple daily injections with basal and bolus analogue insulins	Elective	I, RC, APW, ACD, F Attended workshops 1,2 and remote contribution workshop 3
5	Female	79	White	Type 2 Diabetes	25 years	Self‐managing multiple daily injections with basal and bolus analogue insulins	Elective	I, RC
6	Male	85	White	Type 2 Diabetes	20 years	Self‐managing biphasic insulin three times daily	Emergency	I, RC
7	Female	77	White	Type 2 Diabetes	24 years	Self‐managing basal analogue insulin once daily	Elective	I, RC
8	Female	74	White	Type 2 Diabetes	> 10 years	Self‐managing basal analogue insulin once daily	Elective	I, RC, ACD, F Attended workshops 1, 2, 3
9	Female	91	White	Type 2 Diabetes	> 30 years	Self‐managing intermediate insulin once daily	Elective	I, RC
10	Female	76	White	Type 2 Diabetes	> 10 years	Self‐managing basal analogue insulin once daily	Elective	I, RC

**Staff participants**								
**Study identifier**	**Sex**	**Age (Years)**	**Ethnicity**	**Staff Group & Area of work**		**Professional Experience in NHS (years)**	**SHINE study activities**	
1	Female	30	White	Nursing Registered (Diabetes)		7	I	
2	Female	37	White	Nursing Registered (Surgical ward)		13	I	
3	Female	50	White	Nursing Registered (Frailty)		19	I, ASW, ACD workshop 3, F	
4	Female	48	White	Nursing Registered (Medicines Safety)		30	I, ASW, ACD workshop 2, F	
5	Female	55	White	Nursing Registered (Patient safety)		33	I	
6	Female	29	White	Nursing Registered (Diabetes)		12	I, ASW, ACD Workshop 1, 2, 3, F	
7	Female	55	White	Administrative and clerical (Safety & Governance)		10	I	
8	Female	41	Asian	Medical (Diabetes)		2	I, ASW, F	
9	Female	32	White	Healthcare scientist (Pharmacy)		9.5	I	
10	Female	48	White	Additional clinical services (Surgical wards)		14	I	
11	Male	27	Asian	Medical (Geri‐Orthopaedics)		9 months	I	
12	Female	32	White	Allied health professional (Surgical wards)		7	I	
13	Female	29	White	Nursing Registered (Geri‐Orthopaedics)		11	I	
14	Female	48	White	Nursing Registered (Quality & safety)		24	I, ACD Workshop 1, 2, 3, F	
15	Male	40	African	Medical (Geriatrics)		3	I	
16	Female	26	White	Nursing Registered (Surgical ward)		6	I	
17	Female	34	White	Healthcare scientist (Pharmacy)		12	I	
18	Female	29	White	Nursing Registered (Surgical ward)		8	I	
19	Male	62	Asian	Nursing Registered (Surgical Ward)		20	I	
20	Female	37	Asian	Nursing Registered (Surgical Ward)		8	I	
21	Female	45	White	Medical Registered (Anaesthetics)		21	I	
22	Female	30	Asian	Nursing Registered (Surgical Ward)		4.5	I	
23	Female	57	White	Nursing Registered (Practice Nursing, community)		39	ASW, ACD workshops 1, 2, 3, F	
24	Female	61	White	Allied health professional (Pre‐Op)		22	I, ACD, workshops 1, 2, 3	
25	Female	50	Asian	Nursing Registered (Theatres)		14	I	
26	Female	40	White	Allied health professional (Dietetics)		18	ACD Workshop 1	
27	Female	31	White	Medical (Education)		2	ACD Workshop 2 & 3, F	

*Note:* SHINE study activities: Interview (I); Remote Contribution for patient priorities & co‐design workshop 1 (RC); Attended staff only workshop (ASW); Attended Patient only workshop (APW) Attendance to co‐design workshops (ACD) Feedback of prototypes (F).

### 2.6 Data Analysis

Data were analysed using a process of framework analysis [43, 44, 45] to allow the incorporation of theory and prior knowledge gained throughout the previous phases of the study [19, 21, 36, 46] whilst also allowing new themes to emerge. The analysis process involved the following [43] process:

1. Familiarisation: listening to audio recordings, reading and re‐reading outputs generated at workshops/remote contributions (e.g. transcripts, observational notes, sticky notes, evaluation forms and magic whiteboard).
2. Identifying a thematic framework: An initial framework was developed by the research team through an inductive‐deductive process comprising five categories: patient empowerment, perioperative pathway for people with diabetes; insulin use process tasks in hospital; staff knowledge and confidence; system learning.
3. Coding and Indexing: CLF coded and themed the workshop data and discussed findings with the research team (HH‐A, AF & KW). The framework was refined iteratively through the process.
4. Charting: CLF entered the data into thematic matrices.
5. Mapping and interpretation: Several collaborative analysis sessions by the entire research team reviewing the connections between experiences, challenges, ideas, solutions led to agreement on the final themes and prototypes which are reported in this paper.

#### 2.6.1 Conceptual Model Development

Through an inductive‐deductive process to analyse the interacting components and solution themes and guided by the MRC Framework for intervention development [30], a logic model of a safety intervention was developed [47].

### 2.7 Patient and Public Involvement

A group of older adults living with diabetes (*n* = 10) and their family members (*n* = 2) recruited from an existing peer support group contributed to SHINE study design, and the development of interview topic guides, participant recruitment posters and information sheets [29].

## 3 Results

### 3.1 Participants

All participants were given the option of attending one or more SHINE study activities. In total, there were 37 participants across all the stages of SHINE study. Table 2 provides demographic characteristics of participants and shows stakeholder participation in the different SHINE study activities for inspiration and ideation phases.

For the ideation phase, 10 older adults with diabetes contributed to workshops in the study. All were white, with a median age of 76.5 (range: 68 to 91 years). Seven were female and three were male; four had type 1 diabetes and six had type 2 diabetes. All were self‐managing their insulin treatment prior to hospital admission. Two underwent emergency admissions, and the remainder had elective surgical admissions. Of the 11 healthcare professionals participating across the five workshops, nine were white and two were Asian. Their median age was 42.3 years. Seven were registered nurses, two were doctors, and two were allied healthcare professionals. Their average duration of NHS service was 18 years (ranging from 2 to 39 years).

In terms of contribution, one patient attended the first parallel feedback workshop whilst nine others contributed remotely to the work. One parallel feedback workshop was held for staff with seven healthcare professionals in attendance. Three integrated staff‐patient co‐design workshops were held (*n* = 7, *n* = 8, *n* = 6). Whilst numbers at each face‐to‐face event varied, a total of 10 older adults and 11 healthcare professionals contributed to the co‐design process.

### 3.2 Validation of Touchpoints and Prioritisation of Areas for Intervention (Workshops 1 & 2 & Remote Contributions)

Similar touchpoints originating across different wards at the research site highlighted how patients' experiences were not isolated to specific surgical wards. The areas important to all participants included: the need to identify/acknowledge diabetes at transitions of care; the importance of facilitating self‐administration of insulin in hospital; the need for information to be provided pre‐operatively; and staff confidence with insulin management.

### 3.3 Prioritisation of Areas for Improvement and Intervention (Joint Workshop 3 & Remote Contributions)

The jointly selected areas prioritised for interventions were [1]: Identifying and acknowledging diabetes & insulin at transitions of care [2]; Providing pre‐op advice [3]; Improving patient empowerment in hospital [4]; Right timing and technique of insulin administration in hospital [5]; Enhancing staff knowledge and confidence in diabetes & insulin; and [6] Fostering system learning. A summary of touchpoint categories, participants' parallel and jointly selected priorities, and how these priorities link to the touchpoints across the perioperative care pathway can be found in Appendix 5.

The jointly selected priorities were then analysed by the research team (CLF, HH‐A, AF, KW) and re‐considered using systems thinking and the RESILIENT Framework [21] to develop a conceptual model of an intervention to improve safety. This led to the selection of two important areas for intervention, referred to as ‘*Right insulin, Right time, Right way'* and *‘Safer transitions of care.'* Eight associated actions were identified with these intervention areas by stakeholders. This included: (1) increasing the preparedness of people with diabetes; (2) developing and supporting the workforce; (3) facilitating patient empowerment; (4) increasing accessibility of information; (5) early identification and optimisation of patient insulin management; (6) enhancing systems and practices of insulin use; (7) integrating care pathways; and (8) fostering safe reporting and employing system learning. The joint priorities linked to the areas for intervention and associated actions in the conceptual model can be seen represented visually in Supporting material Appendix 6.

### 3.4 Integrated Workshop 4—Ideation (Solution) Themes

Throughout the process, but particularly in workshop 4, participants identified potential solutions and components for the intervention logic model. These were mapped under six main interlinked themes [1]: It is all so connected [2]; Right insulin, right time, right way [3]; Safer transitions of care [4]; Organisation and provision of care [5]; Empowerment [6]; Supporting and developing staff. Table 3 provides a description of the themes and subthemes, illustrative quotes and their impact on the co‐design process.

**Table 3:** Description of themes including illustrative quotes and ways the themes informed the co‐design process.

Overarching theme & subthemes	Description of theme	Illustrative quotes/excerpts from workshop activities and discussions	Impact on co‐design process and development of SHINE Wheel (Figure 2)
It is all so connected *Overwhelming* *Empathy infused systems approach*	Participants discussed the many interacting components in hospital insulin use, not always considered or captured in insulin safety reviews. Areas where misalignments impacted on insulin safety, within an environment and at a system level were identified. Discussions highlighted how overwhelming it can be to consider the complexity; how to make change and where to start. Participants valued listening to different perspectives and experiences. Participants reflected how a systems approach to explore and learn from insulin use in hospital is still not understood or embedded, yet has potential to increase understanding of complexity of the problem and build system resilience. Empathy and just culture approach needed.	*“It's very complicated, isn't it? And I don't think we can kind of take that away.” (**clinician**, workshop 3)* *“everything's so interlinked.”(**clinician**, workshop)* *“And it's been interesting to hear things from your side because we only see it from our side. And we are of paramount importance to ourselves, but you have to understand where you fit into the bigger picture and how it presents difficulties for you [staff].” (**person with diabetes** workshop 4)* *“if it's reported, or if it's audited, then we find out about it. But if those two things don't happen, then you don't know about it (…) from a bigger sense.” (**Clinician**, workshop 5)* *“There is some really good practice and if we're looking at improving safety and improving system learning, if we can seek to share what went well and replicate the environmental contributory factors that supported that, that's good for everybody.” (**clinician**, workshop 4)*	A systems approach considering interacting components of insulin use [21] informed the development of this study and co‐design process, from selection of activities to the choice of a circular representation for the conceptual model, attempting to relay interconnectivity in components, instead of using a more traditional linear representation. Informed discussions about ideas and solutions. The idea that *hospital insulin safety starts in the community* with empowerment and increasing preparedness of people with diabetes for hospital admission and through key partnerships across the system
Right insulin, right time, right way	Participants identified ways that systems and practices of medications, impacted by multiple interacting components which sometimes misalign, led to insulin use errors. The issue of *right timing* was highlighted by people with diabetes as requiring improvement. Staff identified contributing factors such as systems and processes, knowledge gaps, attitudes and behaviours, accessibility of information. Increasing empowerment ethos, facilitating self‐management could address many insulin administration challenges. The idea of changing nursing processes and starting a “time‐critical drugs medication round” to include insulin was identified to address staff challenges around timely administration of insulin when patients were unable to self‐administer. Safe intravenous insulin infusion management identified as requiring further work to improve staff confidence and safety; streamlining guidelines, training & support, shared decision making.	*“the nursing staff don't understand the importance of timing between insulin and food.” **(person with diabetes**, workshop 2)* *“I think everyone is like treating insulin as the same, like other drugs, not like critical drugs.(…) thinking about when I first qualified (…), I don't think I really recognised the importance of insulin and having it on time” **(clinician**, workshop 1)* *“when I asked for my insulin, because they were bringing round the dinner trolley, and one of the nurses just called out “Well, eat your dinner and then have your injection afterwards.” And I thought, “Well on your head, be it” (laughs) because I know what will happen. But you feel helpless. You know, you can't go running around the ward saying “I want my insulin!” (**person with diabetes** workshop 3)* *Yes, you can discuss the possibility of [intravenous insulin management]. Whose responsibility will it be? And I think that's something that needs to be taken into consideration because it was such a big problem for me.(…) (**person with diabetes** workshop 3)*	This theme holds a central place in the SHINE Wheel, as so many of the challenges participants identified came back to this theme. Specific challenges and ideas on the theme are captured in components of SHINE Wheel; all its actions and outputs link to this theme.
Safer transitions of care *Accessibility of information* *Early identification and optimisation*	Participants identified several challenges associated to transitions of care: Accessibility of information; lack of system and healthcare records integration, communication breakdown Lack of preparedness of people with diabetes (and family members/carers) for hospital admission; assumptions about hospitalisation Characteristics that may be more present in an older population can exacerbate difficulties at transitions of care; Eg. frailty, polypharmacy, cognitive impairment, nutritional deficit, communication barriers, not able to self‐manage Early identification and optimisation and increasing accessibility of information were seen as key to safer transitions of care. The idea emerged to develop standardised diabetes & insulin handovers to ensure better consistency of information at transitions of care; Greater public empowerment and increased preparedness of people with diabetes for hospital admission were considered essential to safer transitions of care; ways to target this identified.	*“Surprising the amount of people who documented “breakdown” in communication for handovers and transition of care” (**clinician**, workshop 1)* *“(…)we had a patient come in on insulin, (…) elderly [with dementia] and the district nurse said they lived out of area, and they didn't know what they were on, the family didn't know the doses, (…) and it was awful, it was a nightmare.” (**clinician**, workshop 5)* *“Yeah, if you don't get it right at the beginning, you're not going to get it right throughout the rest of the journey, are you?” (**Clinician**, workshop1)* *“one of the main problems [listening to patient's stories of admission] was that diabetic patients weren't necessarily aware that they could sign a responsibility, (…) take responsibility for their own care. And for those of us that want to do that it would make a huge difference, (…), it took about five days before I managed to get the form to sign.” (**person with diabetes**, workshop 3)* *“(…) if all of the medications are taken away from the patient, they would assume that now the control is not in my hands so it's the responsibility of the people who have taken my medicines away.” (**clinician**, workshop 1)*	This theme holds a central place in the SHINE Wheel, as so many of the challenges participants identified came back to this theme. Specific challenges and ideas on the theme are captured in components of SHINE Wheel and prototype tools.
Empowerment *Facing the unknown and preparedness* *Facilitating empowerment in hospital*	Improving patient engagement and increased preparedness for hospital admission were identified as key to addressing many of the challenges associated with insulin errors. People with diabetes were often navigating the unknown, feeling a sense of loss of control. Many wished they had received more relevant diabetes & insulin related information prior to admission. Staff identified the need to strengthen empowerment ethos in the hospital to increase facilitated safe self‐management of insulin. The importance of developing partnerships in the community to disseminate key messages to increase preparedness for hospital admission and safer transitions of care was discussed. The idea to provide ‘safety netting' advice in primary care and outpatient dedicated reviews to targeted populations at high risk of hospital admission.	*“Well, it's your life, (…) it's what you've been used to having to keep under control for as long as you've had it. And when that sort of management is not exactly taken away from you, but you haven't got the means to control it, I think that's, well, it's quite damaging.” **(Person with diabetes,** workshop 2**)*** *“Because we take away people's autonomy, don't we, when they come into hospital, completely take it all away and that's wrong.” (**clinician**, workshop 1)* *“we need to know beforehand that we have the right to self‐administer. As soon as we're admitted, we need to know where our insulin is kept. And that the staff on the ward know that you are self‐administering.” **(person with diabetes**, workshop 2)* *“I'm just wondering if patients don't bring their insulin because they just assume we're going to have it, or they don't want it to get lost.” (**clinician**, workshop 5)* *“We were saying about this idea of when people come in [to hospital], they stop being a person, they become a patient and that whole empowerment thing. Like, if we could start that sooner and be like, “Yeah, you are a patient, but you are also someone who manages this” (**clinician**, workshop 5)*	The importance of patient empowerment is weaved into several of the actions and outputs in SHINE Wheel. Specific and detailed ideas and solutions were used in the development of the prototype tools (Supporting Information Appendices 7 and 8) to ensure that these addressed challenges that participants found important on the theme “increasing preparedness for hospital admission”, which also aligns with theme safer transitions of care. Possible partnerships were identified across primary and secondary care to disseminate key messages.
Organisation and provision of care *Dispersed responsibility and lack of joined up thinking* *Communication*	Care pathway integration with patient empowerment at the centre was considered key, as the patient is the sole person who navigates the whole pathway. Participants reflected on how dispersed responsibility and lack of joined up thinking were factors in many insulin errors and significantly affected patient experience and impacted on trust. Disjointed processes and systems within and outside the boundaries of the organisation, knowledge gaps and lack of effective teamwork and communication meant “the patient falls through the gaps”. Technology and systems also play a role as their usability or lack of integration can aid or hinder effective and timely organisation and provision of care.	*“(…) when I saw the surgeon and he put me on the waiting list (…) I asked, (…) about the sliding scale and he said “Oh, you know, that will be sorted out when you come in, that might be up to the anaesthetist. (…), I mentioned it to the anaesthetist, and he said “That's not my concern. All I do is put you to sleep.(…) then you're thinking, “Well, whose responsibility is it?” And it's really frustrating.” (**person with diabetes**, workshop 3)* *“It's a lack of holistic care, isn't it? You work in your specialty and look after this body part and you look after that body part and there's no joined up thinking…” **(clinician**, workshop 3)* *“The person that supports them with their diabetes in primary care isn't the one who's got anything to do with this. They have no idea it's happening until it's all like in a real mess.(…)” (**Clinician**, workshop 1)* *“(…)Your blood sugar is creeping up and up (…) you know that that puts you into an unpredictable regimen (…) You've got all that prior knowledge of how you are affected by that. That makes you more anxious and worried and all results in a lack of trust really, doesn't it in the end? (**person with diabetes**, workshop 3)*	Discussions in workshops highlighted how care integration requires empowerment of patients and staff as well as structural development across the system. Ideas and solutions from participants informed a number of actions and outputs in SHINE Wheel which address aspects of organisation and provision of care; informed the identification of inputs required to the model as well as interacting external, organisational and individual factors; methods of monitoring and evaluation. Discussions informed the elements of context identified in the framework as its multicomponents apply across variable system contexts.
Supporting and developing staff *Staff knowledge and confidence gaps* *Access to support* *Culture*	The importance of supporting and developing staff was recognised at all workshops. Staff knowledge and confidence gaps were a recurrent theme with implications across the perioperative pathway. Access to support was valued, however not always there; over‐reliance on diabetes specialist team. Training opportunities were limited; undergraduate courses did not prepare people sufficiently. Staffing, workload and operational pressures, patient acuity hindered opportunities to develop and support staff and impacted on quality of care delivered. The priority given to diabetes and staff development was not always high. The important role culture plays in improving insulin safety was discussed; a just culture approach needed, however not yet embedded; many participants relayed experiences of a blame culture. Fear of impressions and repercussions hindering system learning.	*“I just, sort of, lay back and thought “Okay, you know what you're doing” without realising that some of them [staff] didn't know what they were up to.” (**person with diabetes**, workshop 3)* *“The lack of knowledge and not feeling confident with insulin [are linked]. If you've got the knowledge about the resources and the guidelines, hopefully there's a bit more confidence in either prescribing or adjusting (…)” (**clinician**, Workshop 1)* *“we've (…) underpinned everything with a just culture approach [design thinking activity in ideation phase]. So, people need to be invested in learning as opposed to blame. (…) if we're pointing fingers at individuals and saying it's because this person had a lack of knowledge, actually, it's very rarely down to one individual's lack of knowledge because we've put them in the position where that's happened. How is the system, you know? And by digging underneath and not (…), you know, pointing fingers at individuals and looking at that broader system, I think we will support people to be much more open and share those experiences in a more thoughtful way, perhaps” (**clinician**, workshop 4)*	The need to address the importance of supporting and development of staff underpins actions and outputs in SHINE wheel. Discussions in workshops informed the identification of inputs required, interacting external, organisational and individual factors, and methods of monitoring and evaluation identified in the conceptual model of the intervention. Prototype tool 2

### 3.5 Integrated Workshop 5—Discussion of SHINE Logic Model

**Figure 2:**
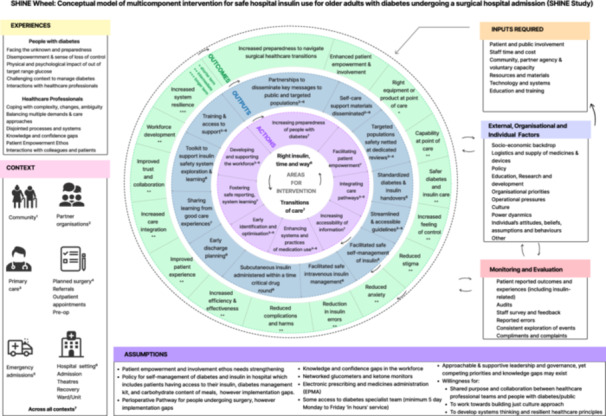
The SHINE Wheel: a conceptual model of intervention developed.

After workshop 4 was analysed, the research team (CLF, HH‐A, AF, KW) prepared an initial prototype of the logic model which contained preliminary areas for intervention, actions, outputs and outcomes. The conceptual model was presented to participants in workshop 5 for validation and refinement, as well as identification of inputs, assumptions, outcomes and monitoring and evaluation methods. The final model, after further participant feedback, can be seen in [Figure 2].

### 3.6 Final Workshop Outputs

#### 3.6.1 SHINE Wheel Logic Model

The SHINE Wheel logic model displays elements of the programme theory graphically as well as contextual dependencies, required inputs, underlying assumptions, and other interacting factors [30]. It also demonstrates the connection between planned work (inputs & actions) and intended results (outputs & outcomes) [47].

It is represented as a wheel to represent the non‐linearity and interconnectivity of its components. The model is conceptualised as a spinning wheel, even spherical, comprising multiple layers with several interacting components.

Two areas for intervention are represented at the centre (‘*Right insulin, Right time, Right way'* and *‘Safer transitions of care')*. Stemming from these, there are eight actions which identify actual changes to be made. These lead to 12 identified outputs which are products/deliverables of the activities. They can be tangible (e.g. a leaflet given to patients) or intangible (e.g. a change to a care process, established or integrated). Sixteen possible outcomes from the intervention model were identified. It also presents the assumptions (underlying beliefs or conditions considered true and necessary for the model) and the input resources that are required (financial, people, estates/equipment, technology etc.) Outcomes are graded from short‐term, intermediate to longer‐term. Seven possible contexts in which areas for intervention, actions and outputs are occurring are identified in the wheel with numbering. The experiences of people with diabetes and healthcare professionals, which underpinned the development of the model, are fully represented. Possible interacting components from an individual, organisational and external environment perspective are also identified.

#### 3.6.2 Co‐Designed Prototypes of Tools to Support Empowerment and Increased Preparedness for Admission

Throughout the workshops, ideas converged to develop prototypes of tools to support patient empowerment, increase patient preparedness for hospital admission and the accessibility of information pre‐operatively. The tools could be used to improve safety for both older and younger insulin users. Professional graphic design services were commissioned to support this process.

##### 3.6.2.1 Tool 1: Five Tips to Increase Preparedness for Hospital Admission

This infographic tool increases the accessibility of relevant information which empowers people with diabetes to prepare appropriately for hospital admission. The target audience are people with diabetes and their family members/carers and can be used across a variety of settings (e.g. in primary care surgeries or as leaflets with dispensed medication). This tool has also been developed into an animation to make it more accessible. [Infographic and hyperlink to animation tool available in Supporting Information Appendix 7].

##### 3.6.2.2 Tool 2: Hospital insulin safety for people with diabetes starts in the community: ways you can help and why

The tool is targeted at healthcare professionals and raises awareness of the problem of insulin errors in hospital and how to mitigate them. The tool is divided into three main areas: why it is important to support people using insulin in hospital; how and why errors happen; and lastly, what action staff can take and what safety netting advice to provide. [Tool available in Supporting Information Appendix 8].

## 4 Discussion

In this study, a process of co‐design and collaboration between older adults with diabetes, staff and researchers culminated in the development of a novel conceptual model for insulin safety innovation. Outputs from the study also included a tool to support patients going into hospital, and another to educate staff looking after them prior to their admission (Appendices 7 and 8).

The use of a partnership approach which fosters collaboration between service users and healthcare professionals to develop meaningful and feasible interventions is well supported by literature [33, 34, 42]. It is also known that older adults are less frequently involved in participatory research methods [48] and that their voice is often absent in the development of insulin safety initiatives. Therefore, our study adopted a collaborative approach to enable older adults and staff to co‐design a conceptual model to support safer hospital insulin use for patients with diabetes undergoing surgical hospital admission.

While many of the model components proposed to improve safety have been identified across different studies [3, 5, 12, 49, 50], the SHINE model is the first known system‐based model to identify priorities and solutions for improving hospital insulin safety as identified by older adults with diabetes and healthcare professionals. As a system‐based model, it advances understanding of the interconnectedness and interaction of multiple aspects of hospital insulin management across the various interfaces and contexts of the perioperative care pathway, including those outside of hospital boundaries [21]. As such, it provides opportunities for clinical leaders, managers and relevant stakeholders to use a systems approach to address hospital insulin safety across the whole care pathway. The need for system approaches to safety, in order to reduce insulin errors in hospital has been previously recognised as important [51, 52].

Within the complex sociotechnical system of healthcare, there are multiple interacting components, unpredictable and emergent behaviours and adaptations, feedback loops and forms of self‐organisation [30, 53, 54]. There is often then, a marked disconnect between work *‘as imagined'* and work *‘as done.'* More recent approaches to patient safety have highlighted the limitations of solely relying on a reactive and regulatory approach to safety and errors (termed Safety‐I). Adopting a Safety‐II approach, which considers both the context in which hospital errors are made and also the actions which actually improve safety, will allow the development of system resilience [54]. In addition, a better understanding of human factors can improve approaches to patient safety [46], and diverse research methods help to understand contextual factors in complex intervention development [30].

Our research identified two key areas for interventions (i.e. *‘Safer transitions of care and Right insulin, Right time, Right way')*. The risk of medication errors at transitions of care has been previously identified [19, 21, 27, 28, 55]. Breakdown in staff communication and inaccessibility of key information at transitions of care have been identified as contributing factors [21, 28, 55]. The SHINE model supports the development of standardised handovers which include relevant information about diabetes and critical medications including insulin. This approach warrants further research but has the potential to facilitate other actions through increasing accessibility of information such as patient self‐management of insulin, HCP awareness of insulin treatment and early optimisation of glycaemia and insulin treatment.

The need for active patient engagement and activation prior to admission has been recognised previously, for example, with the introduction of a perioperative passport for people with diabetes to share key information across services (“*My diabetes passport: planning for surgery*”) [56]. Our study found that lack of preparedness for hospital admission was reported by most participants, hence the development of infographic tools to educate staff and patients (Appendices 7 and 8), may enhance patients' motivation to prepare for admission by using simpler and more direct and active messaging (*this is what you can do type messages*). The intervention model identified in this study also equips patients to navigate the different service transitions throughout their surgical admission and ensure they bring the right equipment or product into hospital to self‐manage.

The three ‘rights' from our data, *right insulin, right time and right way* have long been acknowledged as important in reducing insulin errors [3] and include the importance of appropriate systems and practices which span the whole medication use process [19, 21]. The 'rights' are interconnected and include having the correct product and equipment prescribed and at the point of care (*right insulin*), appropriate insulin administration route (intravenous or subcutaneous) and correct administration technique (*right way*); correct timing of insulin administration, and the appropriate coordination of food and nutritional insulin (*right time*). Time‐critical coordination of tasks, people and equipment is crucial in insulin therapy [21]. Patient engagement and self‐management of insulin in hospital have been identified as a tool to improve the timing of insulin injections [2]. Currently, however, there is unacceptable variation in the use of hospital self‐management policies [3, 18]. Poor communication with older, frailer adults and incorrect assumptions about their self‐care abilities may further exacerbate this issue [57]. Recognising that not all inpatients are able or want to self‐administer insulin is key [11, 12]. Organisations need to have in place appropriate systems to assess and monitor patient desire and suitability for safe self‐administration and management of insulin and to mitigate potential risks [58], but these should not place barriers for engaging patients in self‐management of insulin.

The right timing of insulin administration was particularly important for people with diabetes. Targeted staff education and process changes have been found to improve the timing of insulin administration [5, 59]. For example, staff should be able to recognise that the timing of ‘time‐critical' drugs may fall outside usual drug round times and may need stricter coordination with meals. One recommendation in the SHINE model was to initiate a “time‐critical drug ward round” which includes insulin, recognising that this could also improve patient safety beyond older people with diabetes. Improvements in this area would require adequate staffing levels and changes to culture, processes and systems [50]. Often, diabetes is not the reason for an older person's admission and therefore the staff caring for them may be insufficiently experienced to manage their insulin therapy [7, 51]. There is a clear need for upstreaming interventions such as a greater inclusion of diabetes inpatient care and insulin therapy management in healthcare professional training courses [52].

Finally, several staff members recounted experiences of “*blame”* and others were fearful of consequences of reporting insulin‐related errors [38]. Many considered that review of insulin related incidents focused more on ‘what happened' rather than ‘why' and ‘how' factors and action plans focused frequently on individual/team learning & reflection. Furthermore, there appeared to be limited sharing and critique of learning for effective system change. It was considered that a just culture was still not embedded into the service. To build system resilience, it is recommended that organisations adopt diabetes safety boards with a wide multidisciplinary membership which include regular reviews of hospital insulin safety [7, 51, 60].

### 4.1 Strengths and Limitations

A main strength of this study was that it captured the voice of older adults. When a number of older participants could not attend the workshops, adaptations to methodology (i.e. audio presentation for remote contribution and postal contributions) ensured inclusivity and enabled the views of those with frailty and less mobile participants, or those who disliked group events, still enriched the project process. However, the study has a number of limitations to consider. Research was only conducted in one NHS site, so transferability of our model and prototypes may not be as relevant to other sites either within the UK or internationally. All patient participants were white, which limits the transferability of findings to more diverse populations who may face different cultural or linguistic barriers to insulin safety. Further, more inclusive research is needed, which seeks to engage and involve ethnically diverse populations to understand their specific needs. All patient participants were independent enough to be self‐managing their diabetes and insulin treatment. Further research is needed which includes older adults (and their carers) who cannot self‐manage their diabetes, including people with cognitive impairment, to understand the specific challenges and priorities of these populations when hospitalised, as the current model may not fully address their needs, and they face high risks particularly at transitions of care. By planning more inclusive follow‐up studies which address current limitations and better capture the heterogeneity of the older adult population and their needs, our model and prototypes can be further iterated and expanded, grounded in more diverse perspectives and experiences.

In addition, it was important to acknowledge both that staff outnumbered older adults in workshop attendance and the potential risk of power imbalance between patients and clinical professionals [61, 62]. It was a priority for the research team to ensure that people with diabetes were treated as equal partners across the collaboration and felt heard [62]. This was reiterated at the start of each joint workshop as we recognised the possible power differentials and discussed ground rules. The environment was relaxed, collaborative and inclusive. Evaluation feedback gathered suggested that the older participants felt empowered during the workshops, felt included and were comfortable contributing. Respect, confidentiality, openness and honesty were valued by participants as complex topics such as “blame culture” were discussed sensitively and not shied away from. This led to the identification of the need for willingness to build a just culture approach, as an assumption underpinning the model and the inclusion of fostering safe reporting and system learning as a component within SHINE Wheel.

Agency staff were not included in this study, and therefore, further studies which capture their experience and perspectives are needed.

Finally, the lead researcher (CLF) worked clinically at the research site. Whilst this provided benefits such as familiarity with service and organisational issues, their experience had the potential to affect their facilitation of workshops, analysis and reporting. Efforts were taken throughout to minimise such bias, through using researcher reflexivity techniques (such as keeping a journal, and audit trail of decisions) and regular discussion and critique of results with other research team members (HH‐A, AF, KW) who were external to the hospital site.

## 5 Conclusion

Reducing the incidence of insulin errors in hospital sessions is an ongoing challenge, particularly in the context of older people. This study has introduced a conceptual model of an intervention to enhance hospital insulin safety for older people with diabetes during a surgical admission. The model is grounded in the experiences of patients and health professionals and co‐designed by them. The model can be used to guide the development of future insulin safety interventions. Further studies are needed to refine and test the conceptual model and the tools developed in the study to increase patient preparedness for hospital admission. Areas of future focus for follow up study and refinement of the model and tools should include a more ethnically diverse patient sample, and older adults with cognitive impairment or unable to self‐manage their insulin treatment in hospital.

## Author Contributions

**Christina Lange Ferreira:** conceptualisation, data curation, formal analysis, investigation, validation, methodology, project administration, visualisation, writing original draft, writing – review and editing. **Hellena Habte‐Asres:** conceptualisation, formal analysis, methodology, supervision, validation, writing – review and editing. **Jyothish Govindan:** Methodology, writing – review and editing. **Dionne Mytton:** Investigation, writing – review and editing. **Angus Forbes:** Conceptualisation, formal analysis, methodology, supervision, validation, writing – review and editing. **Kirsty Winkley:** conceptualisation, formal analysis, methodology, supervision, validation, writing – review and editing.

## Ethics Statement

National Health Service (NHS) Health Research Authority ethical approval from East Midlands‐Derby Research Ethics Committee (24/EM/0022) and research site approval was gained prior to study commencement.

## Conflicts of Interest

C.L.F. reports that financial support was provided by the Foundation of European Nurses in Diabetes (FEND). C.L.F. reports having received grants towards research costs from Novo Nordisk UK Research Foundation (NNUKRF), Herefordshire and Mid‐Powis Diabetic Care Fund and Florence Nightingale Faculty of Nursing, Midwifery and Palliative Care Staff Development Fund. H.H.‐A. reports having received speaker honoraria from AstraZeneca and Bayer. K.W., A.F., J.G., D.M.report no conflicts of interest relevant to this article.

## Supporting information

Supporting file 10.2.2026.

## Data Availability

The data that support the findings of this study are available within the article and in the Supporting Material of this article.
