# Supplementary material for: Developing an Intervention for Safe Hospital Insulin Use for Older Adults With Diabetes Undergoing Surgical Admission (SHINE Study): A Co‐Design Study
Source: Health Expect. 2026 Mar 4;29(2):e70622. doi: 10.1111/hex.70622 (PMC12961157; doi:10.1111/hex.70622)

Developing an intervention for **s**afe **h**ospital **i**nsuli**n** us**e** for older adults with diabetes undergoing surgical admission (SHINE Study): A Co-design study

# Supplementary data file

|  |  | Page |
| --- | --- | --- |
| Appendix 1 | Research site characteristics | 2 |
| Appendix 2 | Touchpoints originating from staff interviews | 4 |
| Appendix 3 | Touchpoints originating from interviews with people with diabetes | 6 |
| Appendix 4 | Example of 2x2 matrix completed at integrated staff-patient co-design workshop 2 | 7 |
| Appendix 5 | Perioperative care episode touchpoint summary and prioritisation of areas for intervention development | 8 |
| Appendix 6 | Developing intervention components from participant joint priorities | 9 |
| Appendix 7 | Prototype of Tool 1 developed: Five tips to prepare for hospital stay | 11 |
| Appendix 8 | Prototype of Tool 2 developed: Insulin safety starts in the community | 12 |

## Appendix 1: Research site characteristics

| Research site characteristics | |
| --- | --- |
| Hospital characteristics | District General Hospital in rural area of England. Serving a population of 235000 people, in an area with higher than national prevalence of older adults with diabetes.  The only secondary care hospital in the county, which also serves neighbouring county, in Wales.  Part of a Foundation Group of 4 hospitals across different counties.  The research site has not yet undergone Diabetes Care Accreditation Programme (DCAP). [**DCAP**](https://www.dcap.org.uk/) |
| Diabetes Inpatient Team | Consultant led diabetes specialist inpatient team with diabetes inpatient specialist nurses (DISN) works Monday to Friday 9AM-5PM.  There is a multidisciplinary foot team for diabetes. There is no dedicated inpatient diabetes specialist pharmacist, dietitian or psychologist in post. |
| Perioperative Care | There is a peri-operative pathway based on Centre for Peri-Operative Diabetes Guideline.  There is no Perioperative DSN. |
| Diabetes and insulin safety | Diabetes inpatient team meets weekly to discuss incidents and complaints.  There are quarterly diabetes safety forum meetings in place with patient safety, organizational leads, IT and analytic teams invited where thematic review of incidents and harms are discussed. |
| Guidelines recommended by Joint British Diabetes Societies | The hospital uses guidelines for diabetes hospital care based on the JBDS-IP guidelines, available on the Trust intranet.  There is a guideline in place for emergency alternatives to unavailable insulin preparations. |
| Inpatient self-management of diabetes and insulin | There is a self-management policy for diabetes. |
| Training | Online safe use of insulin e-learning module is mandatory for relevant clinical staff.  Diabetes and insulin safety included in junior doctors’ induction and training. |
| Access to carbohydrate content of meals | Carbohydrate content of hospital meals is available on request. |
| Identification of diabetes on admission and referral to inpatient diabetes team. | There was no electronic system to identify all people with diabetes on admission at the time of the study. EMIS view is integrated with the electronic patient record (EPR) allowing access to GP records, however only available for patients residing in the county.  There is an electronic pathway to refer inpatients with diabetes for inpatient diabetes specialist review. |
| Electronic prescribing systems and support | Electronic prescribing, monitoring and administration (ePMA) is in place with several inbuilt insulin related order sets developed for sub-cutaneous and intravenous (IV) insulin infusion prescribing.  Hospital guidelines guide insulin rate prescribing during IV insulin Infusion use and management. |
| Insulin administration | IV insulin infusions are prepared and set up by nurses in clinical areas guided by hospital protocols. IV insulin monitoring and management is recorded on paper charts at the patient bedside. Bar coded medicines administration is not used. Two nurses conduct independent verification of insulin prescription and device prior to insulin administration. |
| Web-linked blood glucose and ketone meters | Point of care monitoring of capillary blood glucose (CBG) and ketones is undertaken via networked web-linked meters; however, these do not directly upload patient result into EPR. Web-linked networked glucose and ketone levels are monitored by inpatient diabetes specialist nursing team to prioritise in-reach inpatient reviews. |
| Discharge checklist | The hospital has electronic discharge system but there is no specific diabetes discharge checklist in place. |
| Diabetes inpatient harms and participation in audits | The hospital participates in National Diabetes Inpatient Safety Audit (NDISA) reporting patient harms related to hospital acquired diabetic ketoacidosis, hyperosmolar hyperglycaemic state, severe hypoglycaemia and foot lesions/ulcers.  [National Diabetes Inpatient Safety Audit - NHS England Digital](https://digital.nhs.uk/data-and-information/clinical-audits-and-registries/national-diabetes-inpatient-safety-audit)  The hospital submits data to DEKODE-DKA quality improvement project.  [DEKODE—A cloud‐based performance feedback model improved DKA care across multiple hospitals in the UK](https://onlinelibrary.wiley.com/doi/epdf/10.1111/dme.70004) |

## Appendix 2: Touchpoints originating from staff interviews


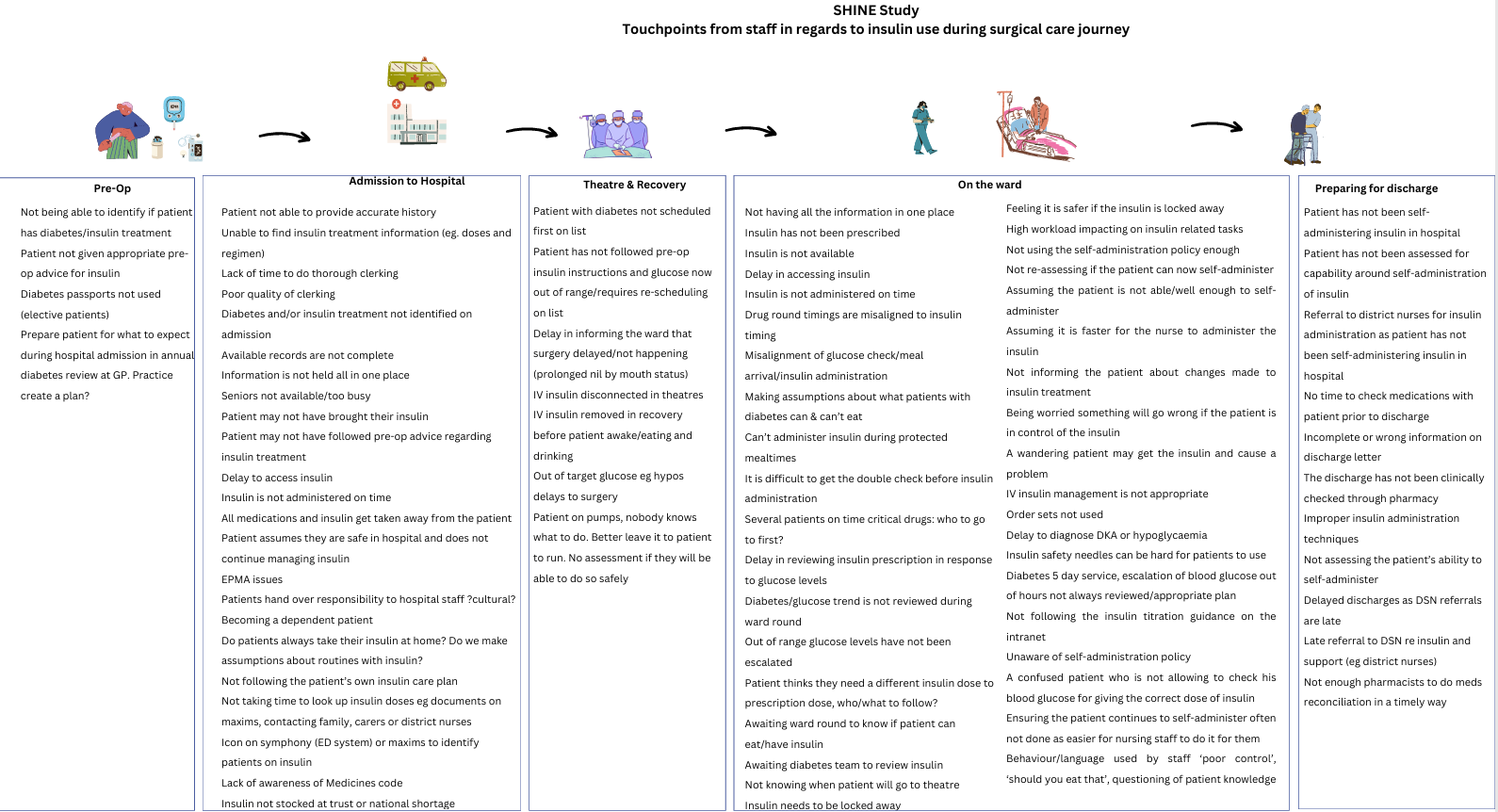


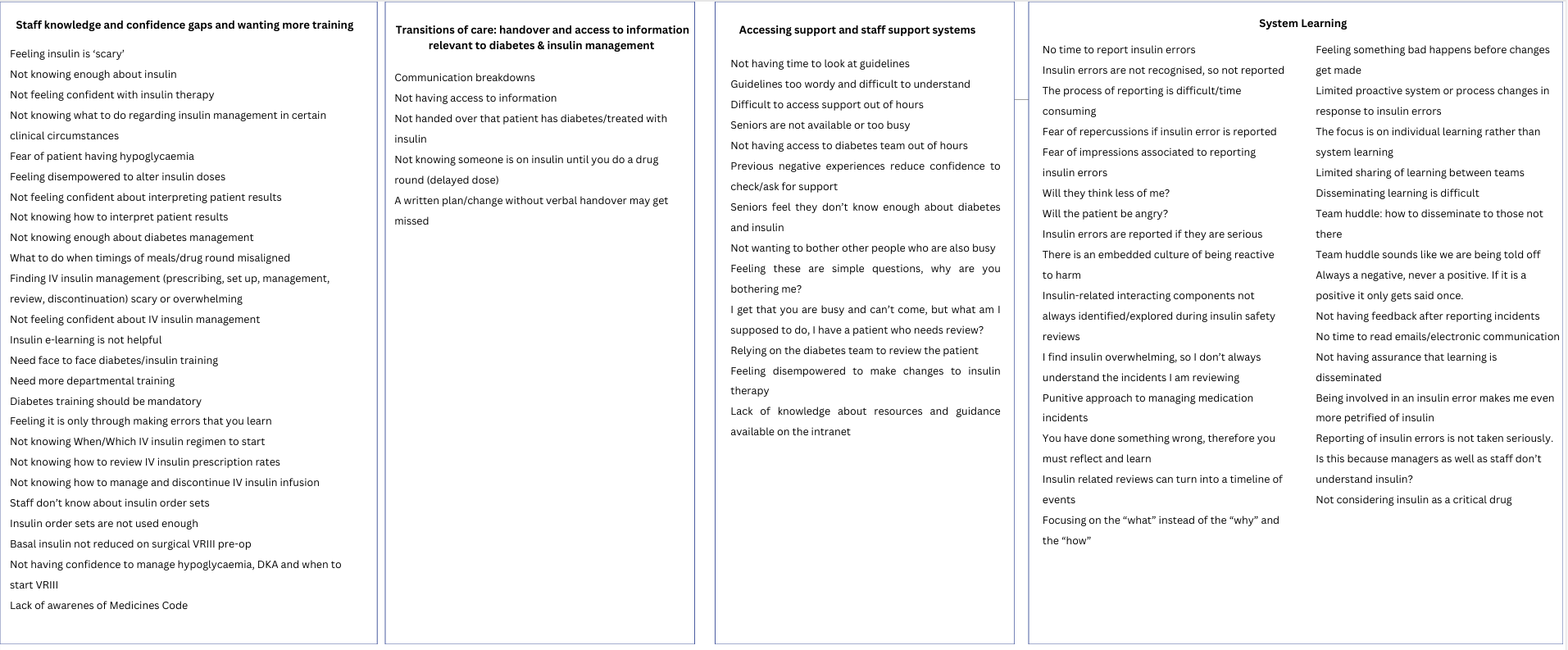


## Appendix 3: Touchpoints originating from interviews with people with diabetes


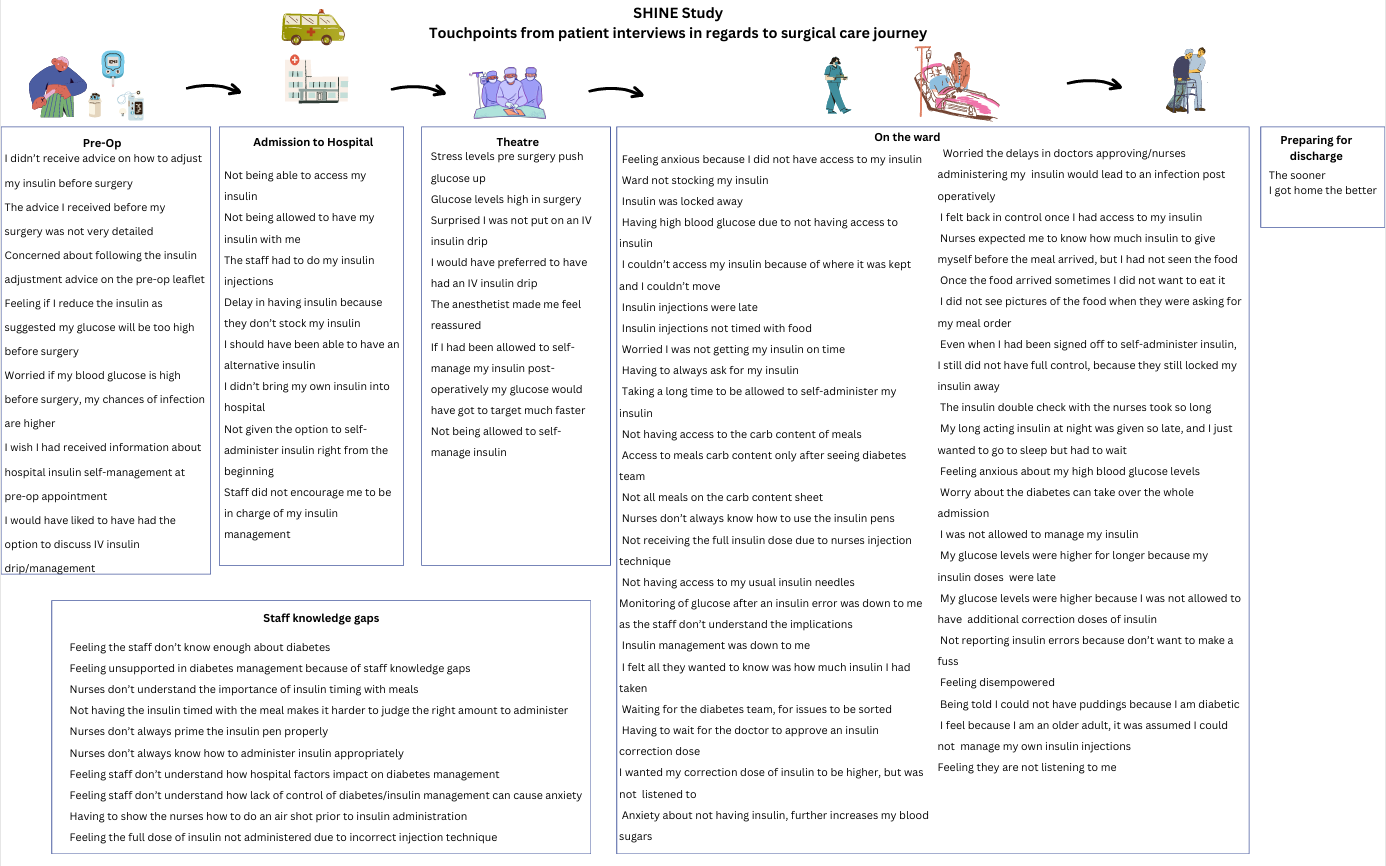


## Appendix 4: Example of 2x2 matrix completed at integrated staff-patient co-design workshop 2


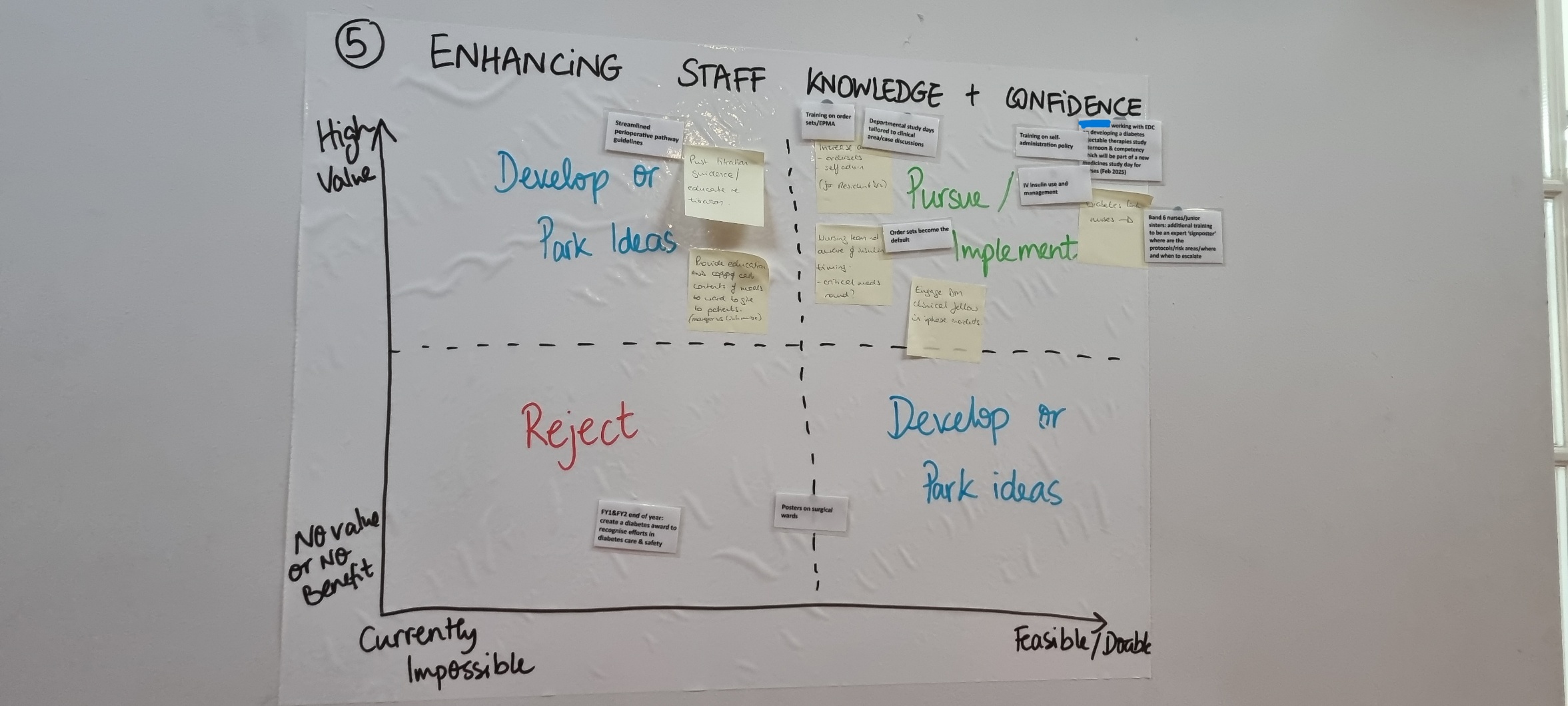


## Appendix 5: Perioperative care episode touchpoint summary and process of prioritisation of areas for intervention development


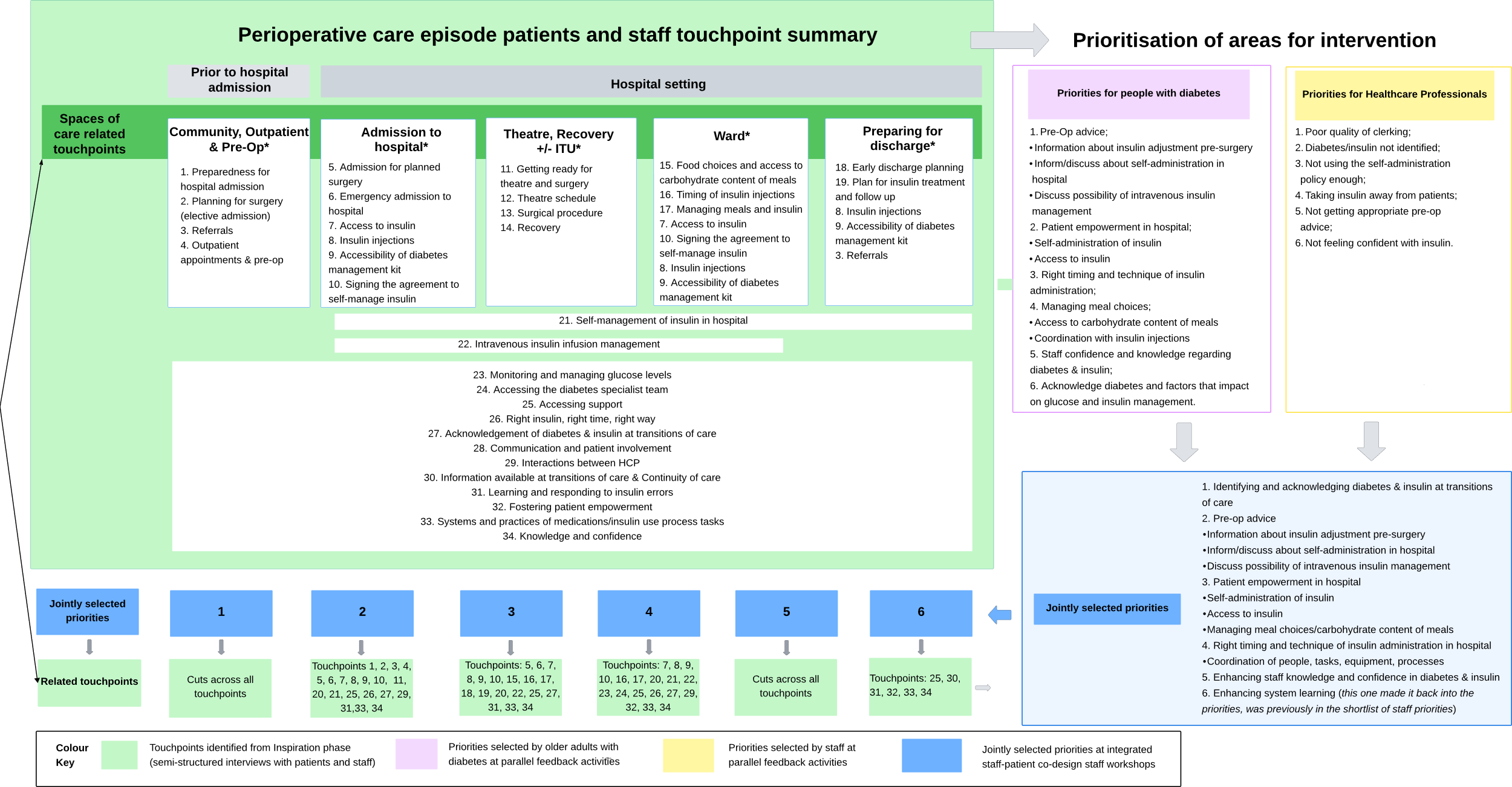


## Appendix 6: Developing intervention components from participant joint priorities

| **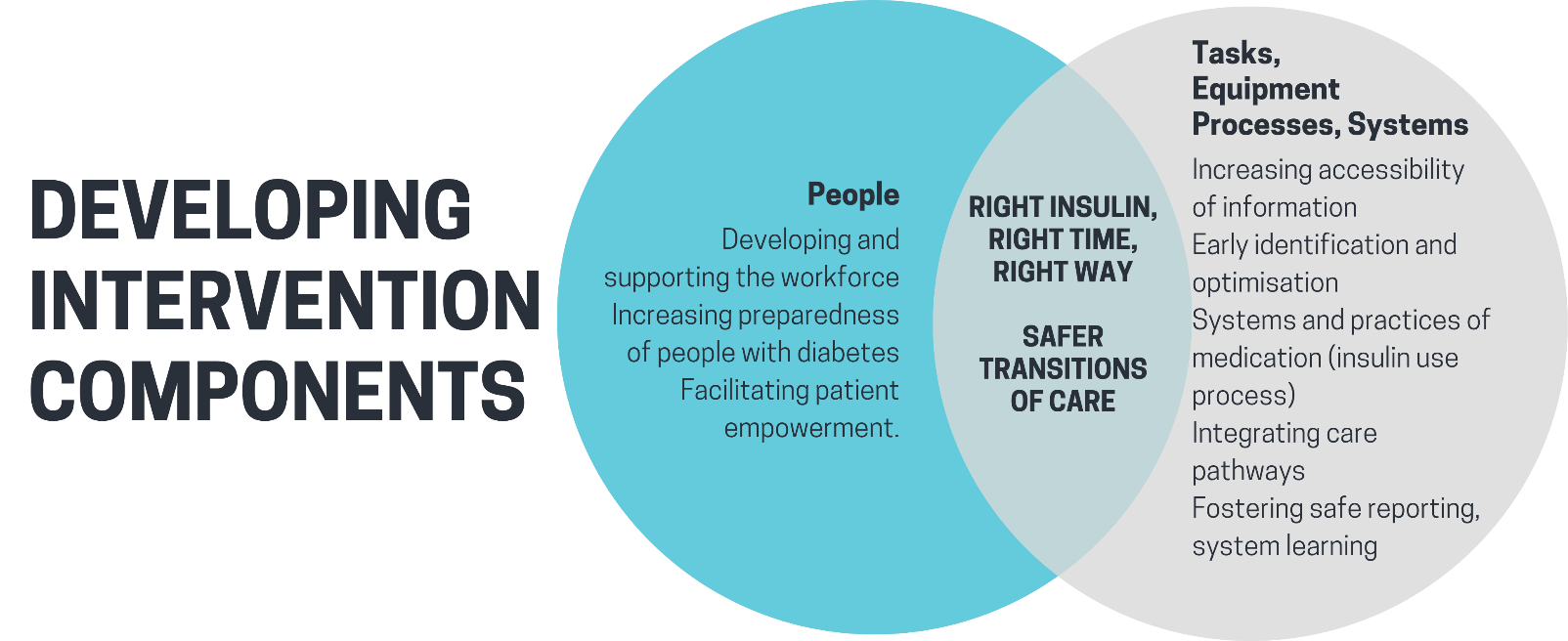**  **Joint priorities linked to areas for intervention and associated actions** | | | |
| --- | --- | --- | --- |
| **Joint Priorities identified by participants** | **Areas for intervention**  **in SHINE Wheel** | **Associated Actions in SHINE Wheel** | **Associated components of RESILIENT Framework (Lange Ferreira et al 2025)** |
| 1. Identifying and acknowledging diabetes and insulin at transitions of care | **Right insulin, right time, right way**  **&**  **Safer transitions of care** | - Increasing accessibility of information - Early identification and optimisation - Systems and practices of medication (including insulin) - Integrating care pathways | - Time critical coordination - Teamwork and communication - Transitions of care - Patient & Person staff - Equipment/Tools - Tasks (including insulin use process tasks) - Internal environment - Organisation - External environment |
| 2. Pre-op advice   - Information about insulin adjustment pre-surgery - Inform/discuss about self-administration in hospital - Discuss possibility of intravenous insulin management |  | - Increasing preparedness of people with diabetes - Increasing accessibility of information - Early identification and optimisation - Systems and practices of medication (including insulin) - Integrating care pathways | - Time-critical coordination - Teamwork and communication - Transitions of care - Patient & Person staff - Equipment/Tools - Tasks (including insulin use process tasks) - Organisation - External environment |
| 3. Patient empowerment in hospital   - Self-administration and management of insulin - Access to insulin - Managing meal choices/carbohydrate content of meals |  | - Increasing preparedness of people with diabetes - Facilitating patient empowerment - Increasing accessibility of information - Early identification and optimisation - Systems and practices of medication (including insulin) - Integrating care pathways | - Time-critical coordination - Teamwork and communication - Transitions of care - Patient & Person staff - Equipment/Tools - Tasks (including insulin use process tasks) - Internal environment - Organisation - External environment |
| 4. Right timing and technique of insulin administration in hospital   - Coordination of people, tasks, equipment, processes |  | - Increasing accessibility of information - Early identification and optimisation - Systems and practices of medication (including insulin) - Integrating care pathways | - Time-critical coordination - Teamwork and communication - Transitions of care - Patient & Person staff - Equipment/Tools - Tasks (including insulin use process tasks) - Internal environment - Organisation |
| 5. Enhancing staff knowledge and confidence in diabetes and insulin |  | - Developing and supporting the workforce - Increasing accessibility of information | - Teamwork and communication - Person staff - Equipment/Tools - Tasks (including insulin use process tasks) - Organisation - External environment |
| 6. Enhancing system learning |  | - Fostering safe reporting, system learning - Increasing accessibility of information | - Time-critical coordination - Teamwork and communication - Transitions of care - Patient and person staff - Organisation - External environment |

Lange Ferreira C, Forbes A, Hashim R, Winkley K. Insulin errors and contributing factors affecting people with diabetes in hospital: A scoping review. Int J Nurs Stud. 2025 Jul;167:105074. doi: 10.1016/j.ijnurstu.2025.105074. Epub 2025 Mar 27. PMID: 40239448.

## Appendix 7: Prototype of tool 1 ‘Five tips’ to prepare for hospital stay


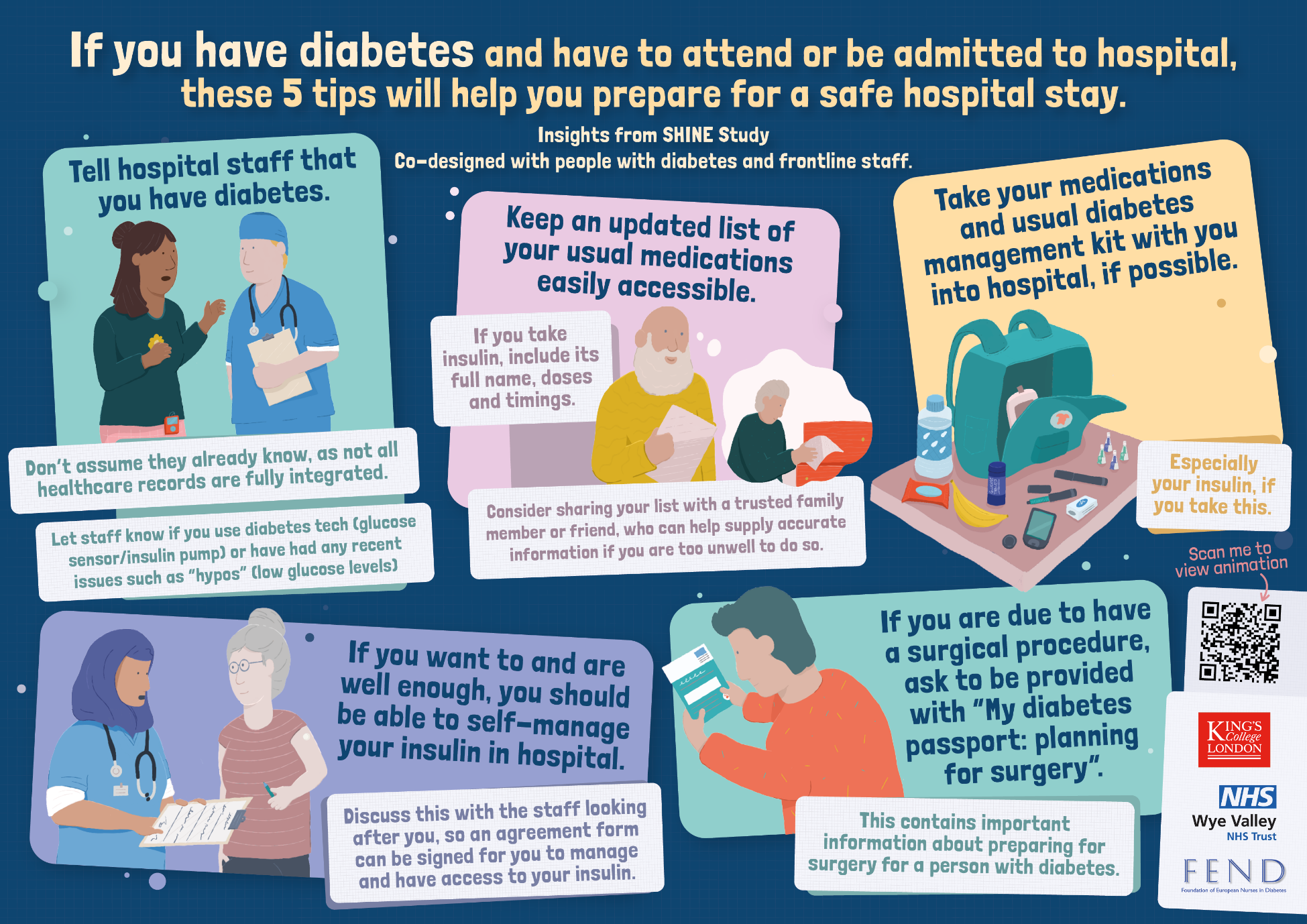


Hyperlink to animation: [Empowering people with diabetes to prepare for a safer hospital stay | 2 min guide | SHINE Study](https://www.youtube.com/watch?v=bAY_FfbfBS0)

## Appendix 8: Prototype of tool 2 Hospital insulin safety starts in the community


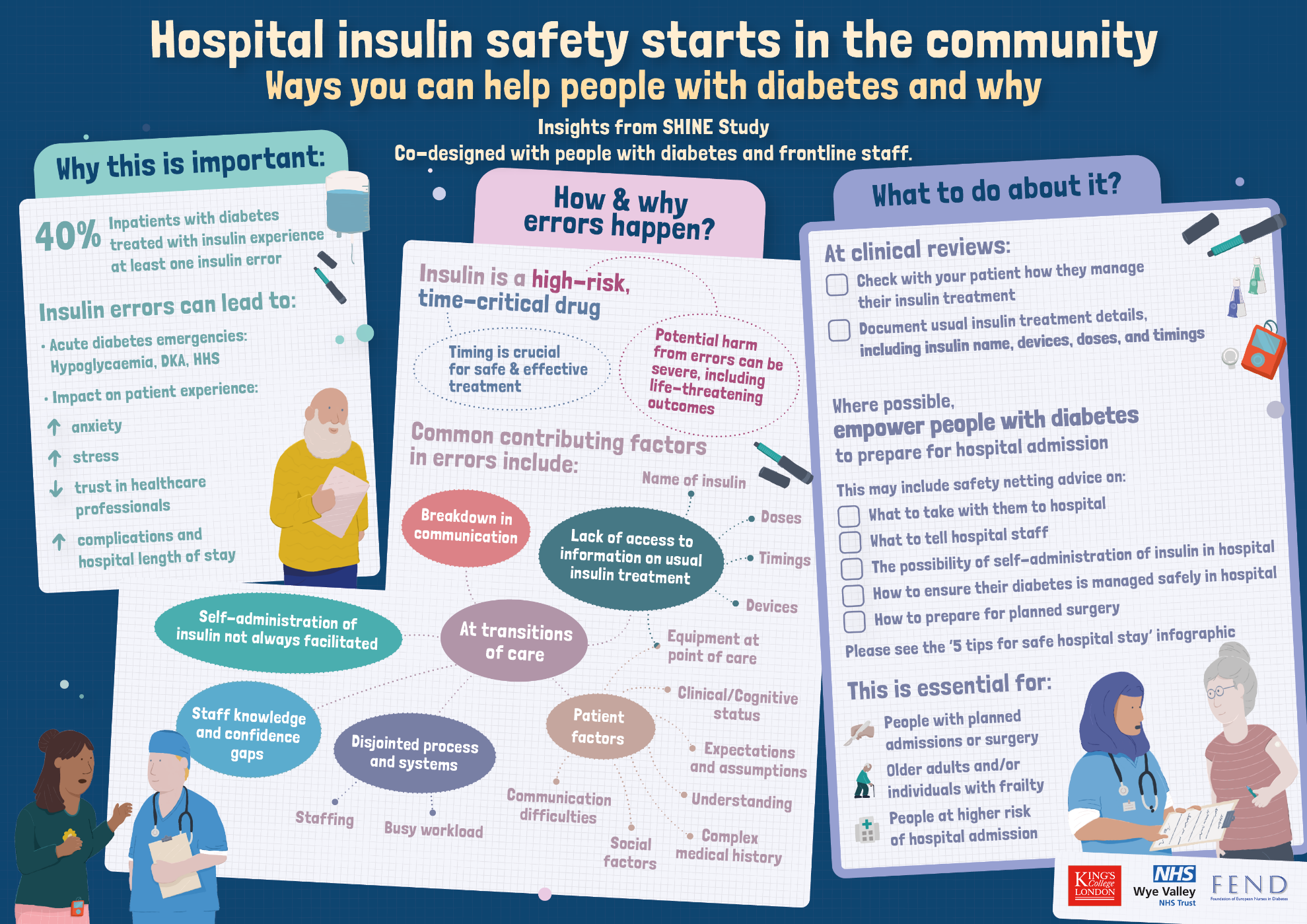

Supplement: Supplementary file 1 — Supporting file 10.2.2026. [file HEX-29-e70622-s001.docx]
